# SENSE OF PURPOSE IN LIFE PREDICTS HIGHER ENGAGEMENT IN COGNITIVELY STIMULATING ACTIVITIES

**DOI:** 10.1093/geroni/igae098.3710

**Published:** 2024-12-31

**Authors:** Nathan Lewis, Kyrsten Hill, Gabrielle Pfund, Payton Rule, Mathias Allemand, Patrick Hill

**Affiliations:** University of British Columbia, Vancouver, British Columbia, Canada; Washington University in St. Louis, St. Louis, Missouri, United States; Auburn University, Auburn, Alabama, United States; Washington University in St. Louis, St. Louis, Missouri, United States; University of Zurich, Zurich, Switzerland; Washington University in St. Louis, St. Louis, Missouri, United States

## Abstract

Several studies have noted associations between having a strong sense of purpose or direction in life and better cognitive health outcomes including better memory, executive functioning, and reduced dementia risk. However, limited research has considered why these associations occur. Engaging in cognitively stimulating activities such as reading is believed to promote cognitive reserve—the brain’s ability to compensate for damage and maintain function even in the face of aging or neurological disease. The present study aimed to test this potential mechanism by examining whether a stronger sense of purpose predicts higher engagement in cognitively stimulating activities, and whether these associations differed based on age and retirement status. Data were collected from an online survey of 787 American adults on Prolific, with over-recruitment of older adults (Mage=50.31, SD=16.34, Range=19-85, 49.30% women, 71.08% White). Multiple regression tested whether those with higher in sense of purpose reported more frequent engagement in 15 activities from the Cognitive Leisure Activity Scale (e.g., reading a book/newspaper, doing puzzles, attending a club or group activity). Having a stronger sense of purpose in life was associated with more frequent participation in cognitive leisure activities (β=0.25, 95% CI [0.19, 0.32], p<.001) adjusting for age, gender, race, income, and retirement status. Additional models found no significant interactions with age (β= 0.00, 95% CI [-0.00, 0.01], p=.156) and retirement status (β=0.09, 95% CI [-0.09, 0.24], p=.236). These findings suggest that a strong sense of purpose may encourage cognitive activity, regardless of age or retirement, potentially supporting cognitive health.

